# Case Report: A Variant Non-ketotic Hyperglycinemia With *GLRX5* Mutations: Manifestation of Deficiency of Activities of the Respiratory Chain Enzymes

**DOI:** 10.3389/fgene.2021.605778

**Published:** 2021-05-13

**Authors:** Wei-xing Feng, Xiu-wei Zhuo, Zhi-mei Liu, Jiu-wei Li, Wei-hua Zhang, Yun Wu, Tong-li Han, Fang Fang

**Affiliations:** Neurology Department, National Center for Children's Health China, Beijing Children Hospital Affiliated to Capital Medical University, Beijing, China

**Keywords:** variant non-ketotic hyperglycinaemia, glutaredoxin 5, stop codon, compound heterozygous mutations, pediatric patient

## Abstract

**Objective:** Variant non-ketotic hyperglycinaemia (NKH) is a rare disorder characterized by variable clinical, biochemical, and imaging features. The variant form of NKH is rare and characterized by variable clinical, biochemical and imaging features.

**Subjects:** Herein, we report a girl with variant NKH with two mutations in glutaredoxin 5 (*GLRX5*), which has been described in only three patients.

**Results:** The clinical and biochemical phenotypes of the patient are also described. She suffered from developmental regression associated with spasticity, developmental delay, anemia and optic atrophy. The mitochondrial leukoencephalopathy was used to designate these disorders. An increased T2 signal from the medulla oblongata to the C6 spinal region was also observed on spinal cord MRI. Tandem mass analysis of a dried blood sample revealed elevated levels of glycine. The patient has two compound heterozygous mutations (c.151_153 del AAG and c.196C>T) in the *GLRX5* gene. The c.196C>T mutation led to a stop codon (p.Q66Ter). Activities of mitochondrial respiratory chain (MRC) complexes II+III in the patient's fibroblasts were abnormal.

**Conclusions:** We present the case of a girl with variant NKH who manifested spasticity and bilateral cavitating leukoencephalopathy. The patient had a deficiency of a respiratory chain enzyme, and this is the first report. Genetic testing is important for physicians to evaluate suspected variant NKH patients and to provide proper genetic counseling.

## Introduction

Non-ketotic hyperglycinaemia (NKH) is a disorder that affects the mitochondrial glycine cleavage enzyme system. NKH is characterized by elevated glycine in serum, cerebrospinal fluid and other body compartments. It can be classified into classic (or typical) and variant (or atypical) forms (Dinopoulos et al., 2005). Classic NKH occurs in the neonatal period; severe infantile encephalopathy, hypotonia, seizures, and progressive apnoea are the major symptoms. Considerably elevated glycine levels in the plasma and cerebrospinal fluid (CSF) of these patients have been observed (Poothrikovil et al., 2019). Approximately 70~75% of classic NKH patients carry mutations in the *GLDC* gene, whereas 20% have mutations in *AMT*, and 1% have mutations in *GCSH* (Toone et al., 2001). The variant form of NKH is rare and characterized by variable clinical, biochemical and imaging features. These cases differ in the age of onset and presentation. Deficiencies in the biosynthesis of lipoyl-H lipoate synthase (*LIAS*), glutaredoxin 5(*GLRX5*), and BolA type 3(*BOLA3*) can cause variant NKH (Kikuchi et al., 2008). A novel homozygous missense mutation in the *GLDC* gene of an atypical NKH patient has also been reported (Baker et al., 2014).

Only three variant NKH patients have been reported to carry mutations in *GLRX5*, as described by Chiong et al. (2007), Wei et al. (2011), and Baker et al. (2014). (*GLRX5*; OMIM 616859). The three cases were two Lebanese girls (onset ages, 2.5 years and 7 years) and one Chinese boy in Taiwan (onset age, 2.5 years). In the two Lebanese girls, *GLRX5* exhibited an exon 1,c.151_153delAAG; p.K51del, homozygous deletion. In the Chinese boy, the mutation c.151_153delAAG was found on the maternal allele, and the 8-bp insertion c.82_83insGCGTGCGG; p.G28Gfs*25 was found on the paternal allele, resulting in a premature stop codon at amino acid 52, located 25 amino acid residues downstream. Patient samples were genotyped using Affymetrix SNP 6.0 chips. Herein, we report a Chinese girl with variant NKH with mutations in *GLRX5*. She suffered development retrogression associated with spasticity and developmental delay. Unfortunately, she died of respiratory failure after the infection. MRI showed signal abnormalities involving the bilateral cavitating leukoencephalopathy and long tract-like lesions of the spinal cord. Mutations in *GLRX5* were detected, namely, c.151_153 del AAG [p.K51del] (of maternal origin) and c.196C>T [p.Q66Ter] (of paternal origin). The c.196C>T [p.Q66Ter] mutation was characterized as a stop codon and protein truncation. This mutation is pathogenic.

## Patient and Result

**Figure 1:**
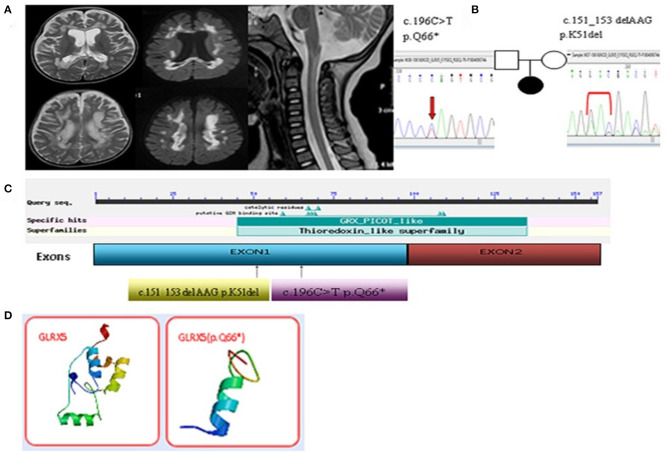
**(A)** Enlarged lesions in the frontal, parietal and occipital of the white matter and the corpus callosum in T2 and the diffusion weighted images were observed. Spinal MRI images showed central lesions of the upper spinal cord. **(B)** Two compound heterozygous missense mutations were identified in the GLRX5 gene. **(C)** The mutation sites of the GLRX5. **(D)** The difference of the noted stop codon c.196C>T[p.Q66*] of the GLRX5 protein in the three-dimensional modeling prediction.

The girl was Han Chinese in Shandong Province, and she was born normally. Her developmental history was also standard until 1 year and 3 months of age. She was able to sit steadily, crawl, stand with support, and say “mum” and “dad.” Then, the patient began to exhibit clumsy and uncoordinated movements. She was subsequently admitted to the local hospital. Multifocal lesions were detected on MRI by increased signal intensity on the T2 sequence of the bilateral corona radiata, centrum ovale, and corpus callosum. The cystic lesions were observed in the abnormal white matter (Figure 1A). Besides, increased signal intensity in the frontal, parietal and occipital white matter was noted on T2-weighted and diffusion-weighted imaging. The bilateral cavitating leukoencephalopathy was used to designate these disorders. Tandem mass analysis of a dried blood sample revealed elevated levels of glycine (812 μM vs. normal 20–760 μM). No treatment was administered initially at the local hospital to which she was admitted. However, her condition deteriorated after 2 months, with difficulty in movements and language ability. She was unable to raise her head or sit steadily. She presented with stiffness and spasticity in all extremities. She only screamed and no longer said “mum” and “dad.” At this point, she was admitted to our hospital. She suffered from slight encephalopathy, was irritable, and had been febrile for 8 days. Neurological examination showed spastic paralysis of the four extremities, reduced muscle strength (3 to 4 degrees), clasp-knife-like muscular tension, hyperactivity of the tendon reflexes, bilateral clonus, ankle clonus, and the Babinski sign. Several blood tests showed below-normal hemoglobin levels (Appendix 1), which suggested hypochromic microcytic anemia. She suffered from intermittent mild anemia after 6 months of age, but her parents did not attend to it. The cytology test of cerebrospinal fluid (CSF) and CSF total protein were normal. Routine blood biochemical examination revealed elevated blood lactic acid (3.47 mmol/L, normal 2 mmol/L) and increased CK (457 U/L) levels. The urinary organic acid analysis and dried blood spot analysis using GC/MS and tandem mass analysis showed normal results.

Re-examination by MRI revealed enlarged lesions in the frontal, parietal and occipital regions of the deep white matter and the corpus callosum, and the cystic lesions also were observed in the abnormal white matter. Encephalatrophy was observed in the cerebrum and cerebellum. Spinal MRI indicated an increased T2 signal from the medulla oblongata to the C6 spinal region (Figure 1A). The MRA, MRV and SWI were normal. Slow waves were detected by electroencephalography (EEG). Leopard skin-like fundus oculi and bilateral optic atrophy were observed in the ophthalmological examinations.

As the girl had been febrile for 8 days. We provided the treatment of acyclovir and ceftriaxone, as we can't exclude central nervous system infections in the early stage of hospitalization. And patient was treated with baclofen, nitrazepam and trihexyphenidyl were administered for spasticity. She was diagnosed with mitochondrial disease. Vitamins B1, B2, C, and E, coenzyme Q10, biotin and iron dextran were prescribed. On day 21, she was released from the hospital with a slightly improved condition. Her muscular tension was slightly decreased. The clinical symptoms, MRI imaging and next-generation sequencing (NGS) result provided important evidence for the diagnosis of the variant non-ketotic hyperglycinaemia. Lipoic acid and 5 ALAs were administered for treatment. We then followed up with the patient's family until now. The patient's condition showed improvement in terms of her intelligence, and she once again could say “mum” and “dad” when she was followed up for 1 and 1 and a half year. However, she died of infection when 4 years old in the local hospital.

We collaborated with SINOPATH Medical Inspection Inc. to perform whole-exome sequencing utilizing NGS with the proposita sample on Hiseq X (Illumina, CA). Then, Sanger sequencing was performed to confirm the findings of NGS. Sequencing data were aligned to the human genome reference. Next Gene V2.3.4 software was used to evaluate the coverage and mean read depth of the target regions and to identify variants. The mean read depth was 151.24 × 97, and 95% of the target regions covered at least 20x. Two point mutations of *GLRX5*, c.151_153 del AAG [p.K51del] (of maternal origin) and c.196C>T [p.Q66Ter] (of paternal origin), were found in the DNA of the proband (Figures 1B,C and Appendix 2). The parents were healthy. This c.196C>T mutation led to a new stop codon that was found to be deleterious via *in silico* analysis by SIFT, PolyPhen and Mutation_Taster software programs. The position of the c.196C>T residue was conserved among different species (http://genome.ucsc.edu/index.html). The p.K51del and p.Q66Ter is located in the thioredoxin superfamily domain. Figure 1D indicates the difference in the p.Q66Ter of the GLRX5 protein in the three-dimensional modeling prediction (https://swissmodel.expasy.org/). This suggests a pathogenic role for the non-sense variant resulting in a premature stop codon and protein truncation.

Skin fibroblasts from the patient and control fibroblasts (fHDF, Toyobo) were cultured in Dulbecco's modified Eagle medium (DMEM; Gibco^®^, Life Technologies, Thermo Fisher Scientific) in the presence of 1% penicillin/streptomycin and 10% fetal bovine serum (both Gibco^®^, Life Technologies, Thermo Fisher Scientific) at 37°C and 5% CO_2_. Activities of mitochondrial respiratory chain (MRC) complexes I, II, II+III, III, IV, and the mitochondrial marker enzyme citrate synthase (CS) were assayed in isolated mitochondria obtained from skin fibroblasts, as described previously (Kirby et al., 1999; Frazier and Thorburn, 2012; Ogawa et al., 2017). Enzyme activities of each complex were presented as the percentage of normal control mean relative to appropriate reference enzyme activities, such as citrate synthase. Oxygen comsumption rate (OCR) was measured in the patient's cultured fibroblasts with XF96 Extracellular Flux Analyzer (Seahorse Bioscience, Billerica, MA, USA). Samples were prepared as reported (Ogawa et al., 2017, Kremer and Prokisch, 2017).

Activities of MRC complexes II+III in the patient's fibroblasts was abnormal (CS ratio 37%), and complex II and complex III were mild declines, CS ratio 47.3% and 41.3, respectively. MRR (maximum respiration rate) was significantly lower than the control in glucose-containing and galactose-containing medium, respectively (37 and 33% of the control, respectively). Basal OCR also decreased compared with the control (Appendix 3). MRR was expressed as a percentage relative to the average of controls, in which a reduction to <71.6% is considered to represent a significant decline (Ogawa et al., 2017).

## Discussion

Non-ketotic hyperglycinaemia (NKH) is an autosomal recessive disorder in the glycine cleavage system (GCS), which is a multienzyme complex located in the inner mitochondrial membrane of the brain, liver and kidney. The variant NKH patients have less severe presentations, variable ages of onset, and milder neurological symptoms than classic NKH (Hiraga et al., 1981; Kremer and Prokisch, 2017). Non-ketotic hyperglycinemia (NKH) is a well-recognized metabolic cause of life-threatening illness in the neonate. The variant NKH most patients with normal development for 6 months. Then they lost all milestones gradually, and often signifies a poor prognosis (Baker et al., 2014). In this case, the girl died of respiratory failure after the infection which may be associated with deficiency of activities of the respiratory chain enzymes. It was not reported before.

Diagnosing variant NKH is more difficult than diagnosing the classic form because the clinical manifestations are non-specific. Variant NKH may be diagnosed according to the developmental retrogression associated with spasticity and the MRI. Our MRI findings matched the pathology of variant NKH. The white matter involvement was recognized as a manifestation of mitochondrial disorders and the cavitating leukoencephalopathy were used to designate these disorders. They are mainly defined by the MRI characteristics such as cystic lesions in the abnormal white matter (Bindu et al., 2018). In our case, the brain MRI revealed the coincident and typically high T2 signal and diffusion restriction in the frontal, parietal and occipital white matter, as well as the corpus callosum and spine. These findings matched the pathology of variant NKH. The cranial MRI of the Lebanese girl also revealed abnormalities in the frontal and parietal white matter, which then progressed to the deep white matter, periaqueductal gray matter, and around the central canal in the cervical spine (Chiong et al., 2007). The variant non-ketotic hyperglycinaemia patients can have defective glycine transport systems in both the brain and the spinal cord. Glycine transporters may play an important role in brain and spinal cord function. Lipoate is an essential co-factor for the glycine cleavage system, and lipoate biosynthesis requires lipoate synthase enzyme (LIAS), which need [Fe-S]. It has therefore been speculated that the *GLRX5* mutation may impair [Fe-S] transfer to the LIAS protein. The subcortical white matter and the genu of the corpus callosum were the regions with the highest lesion burden (Liu et al., 1993; Zafra et al., 1995).

Our patient showed reduced activities of MRC complexs II+III, and mild decline of complex II and complex III. Iron-sulfur clusters are incorporated in many enzymes including respiratory chain complexes I, II and III and the aconitases (Baker et al., 2014). In theory, deficiency in Iron-sulfur clusters can have reduced activities of these respiratory chain enzymes, and some previously reported BOLA3 mutation patients had reduced activities of some respiratory chain enzymes (Cameron et al., 2011; Haack et al., 2013). None of the reported *GLRX5* mutation patients had a deficiency of a respiratory chain enzyme, and this is the first report. And the girl died of respiratory failure which may be associated with it.

Mutations in *GLRX5* have been associated with the variant NKH in humans because of disorders in the biosynthesis of lipoyl-H. *GLRX5*, c.151_153 del AAG [p.K51del] (of maternal origin) and c.196C>T [p.Q66Ter] (of paternal origin) were detected in the patient. The stop codon, c.196C>T [p.Q66Ter], which was not reported in the Human Gene Mutation Database, and it was found to be deleterious *in silico* analysis. This may lead to a poor prognosis. The deleted lysine 51 lies in the highly conserved glutaredoxin domain (Baker et al., 2014). The mutation c.151_153 delAAG [p.K51del] in *GLRX5* was also discovered in two Lebanese girls and the Chinese boy. It was reported that cells expressing the K51del mutation had decreased PDH and alpha-KGDH complex activities (Liu et al., 2016). The gene *GLRX5* encodes a mitochondrial protein, which is evolutionarily conserved. It is involved in the biogenesis of iron-sulfur clusters, which are required for normal iron homeostasis. In this case, the mitochondrial respiratory chain enzyme defect was also confirmed.

This study reported a case of variant NKH with the *GLRX5* mutation. Mutations in *GLRX5* have been reported in sideroblastic anemia as well as variant non-ketotic hyperglycinaemia in humans (Liu et al., 2014; Ogawa et al., 2017). In this case, intermittent mild anemia was observed at 6 months of age, and hypochromic microcytic anemia was confirmed by blood tests (Appendix 1). These symptoms were associated with the *GLRX5* mutation (Lill et al., 2012). GLRX5 is a mitochondrial protein that plays an essential role in mitochondrial iron-sulfur cluster transfer. Interestingly, Gang Liu reported a Chinese congenital sideroblastic anemia patient with two compound heterozygous missense mutations in *GLRX5*(c.301A>C;K101Q and c.443T>C;L148S). GLRX5 plays a major role in mitochondrial [Fe-S] transfer to apoproteins.The heme biosynthesis pathway is more vulnerable to GLRX5 dysfunction.And that patient did not exhibit central nervous system symptoms. Thus, different mutations, even though they occur in the same *GLRX5* gene, may manifest different phenotypes (Liu et al., 2014).

As a limitation, we did not get the data on glycine determination in CSF. And this case may be described little information as one patient (No.31) in 37 patients diagnosed with cavitating leukoencephalopathies (Zhang et al., 2019). That report was an excellent study. However, it focused on 37 patients. It's unrealistic to describe each patient in detail. And we provide explicit clinical information and follow-up of the patient. Importantly, we found the activities of MRC complexes II+III in the patient's fibroblasts was abnormal and complex II and complex III were mild declines.

In conclusion, an association between *GLRX5* gene mutations and variant NKH has been identified and described. The c.196C>T mutation was identified as a stop codon which may associate a poor prognosis. This is the first report of the deficiency of activities of the respiratory chain enzymes of the patient with *GLRX5* mutations. It is of great importance to characterize any suspected cases at the genetic level to enhance the awareness of variant NKH.

## Data Availability Statement

The raw data supporting the conclusions of this article will be made available by the authors, without undue reservation.

## Ethics Statement

The studies involving human participants were reviewed and approved by the Ethics Committee of Beijing Children's Hospital. Written informed consent to participate in this study was provided by the participants' legal guardian/next of kin. Written informed consent was obtained from the individual(s), and minor(s)' legal guardian/next of kin, for the publication of any potentially identifiable images or data included in this article.

## Author Contributions

FF and W-xF: conceived and designed the manuscript. X-wZ, J-wL, and W-hZ: clinical data acquisition. T-lH, YW, and Z-mL: analyzed the clinical and genetic data. W-xF, FF, and YW: wrote the paper. All authors contributed to the article and approved the submitted version.

## Conflict of Interest

The authors declare that the research was conducted in the absence of any commercial or financial relationships that could be construed as a potential conflict of interest.
