# Supplementary material for: Case Report: A Variant Non-ketotic Hyperglycinemia With GLRX5 Mutations: Manifestation of Deficiency of Activities of the Respiratory Chain Enzymes
Source: Front Genet. 2021 May 13;12:605778. doi: 10.3389/fgene.2021.605778 (PMC8155699; doi:10.3389/fgene.2021.605778)
Supplement: Supplementary file 1 [file Table_1.DOCX]

**Appendix 1. Laboratory investigations of the patient**

| **Laboratory investigations** | | | | | | |
| --- | --- | --- | --- | --- | --- | --- |
| **Routine blood test** | **2018.2.28** | **2018.3.4** | **2018.3.9** | **2018.3.12** | **2018.3.16** | **2018.4.22** |
| HB（g/L）(110-160)* | 107 | 81 | 99 | 97 | 99 | 117 |
| HCT（%）(35-55)* | 33.3 | 26 | 32.3 | 31.9 | 33.1 | 36.8 |
| MCV（fl）(80-100)* | 69.1 | 72 | 73.2 | 75.2 | 78.3 | 74.6 |
| MCH（pg）(27.4-34)* | 22.2 | 22.4 | 22.4 | 22.9 | 23.4 | 23.7 |
| MCHC（g/L) (320-360)* | 321 | 312 | 307 | 304 | 299 | 318 |
| **Blood Biochemic** |  |  |  |  |  |  |
| CK（U/L) (25-200)* | 457 | 81 | 253 | 277 |  |  |
| CK-MB（U/L) (5-40)* | 29 | 34 | 42 | 43 |  |  |
| AST（U/L) (5-40)* | 44.2 | 32.3 | 58.7 | 74.7 |  |  |
| **Blood Lactate**（0.5-2.2)* | 3.47mmol/L |  |  |  |  |  |
| **Blood Ammonia**(18-72)* | 53umol/L |  |  |  |  |  |
| **Glycine** | 812.9uM  (20-760)* | 235.2uM  (45-236)* |  |  |  |  |

*The normal range; HB [haemoglobin](https://fanyi.so.com/#haemoglobin); HCT hematocrit; MCV mean corpuscular volume; MCH mean

corpuscular hemoglobin; MCHC mean corpuscular hemoglobin concentration; CK creatine kinase; CK-MB creatine kinase isoenzymes; AST aspartate transaminase;
