# Supplementary figures and images for: Case Report: A Variant Non-ketotic Hyperglycinemia With GLRX5 Mutations: Manifestation of Deficiency of Activities of the Respiratory Chain Enzymes
Source: Front Genet. 2021 May 13;12:605778. doi: 10.3389/fgene.2021.605778 (PMC8155699; doi:10.3389/fgene.2021.605778)

**Glucose media**


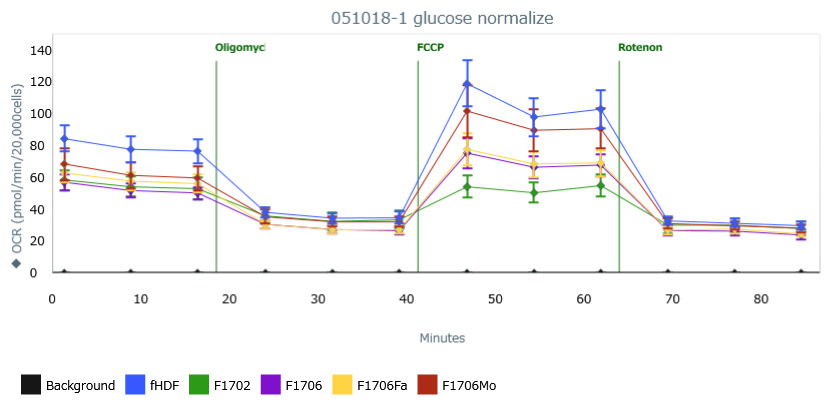


fHDF：Control, F1702：Patient


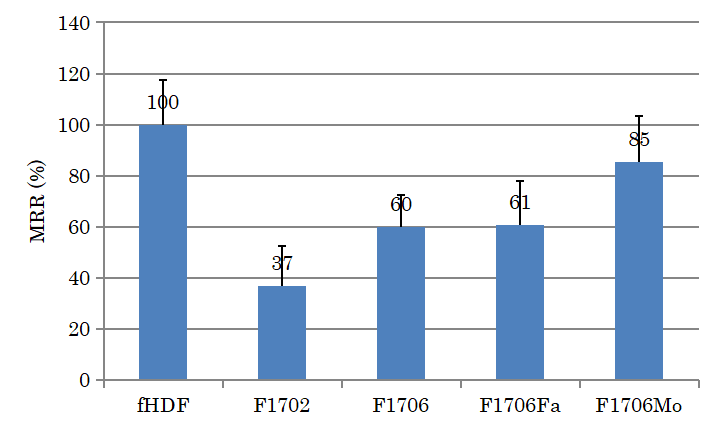


**Galactose media**


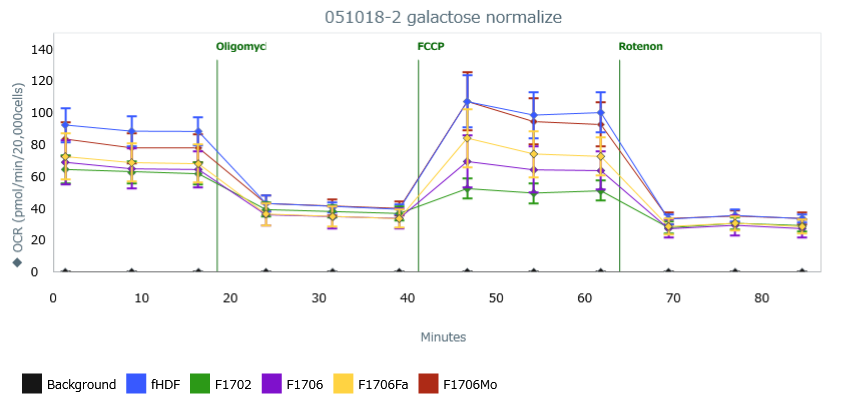


fHDF：Control, F1702：Patient


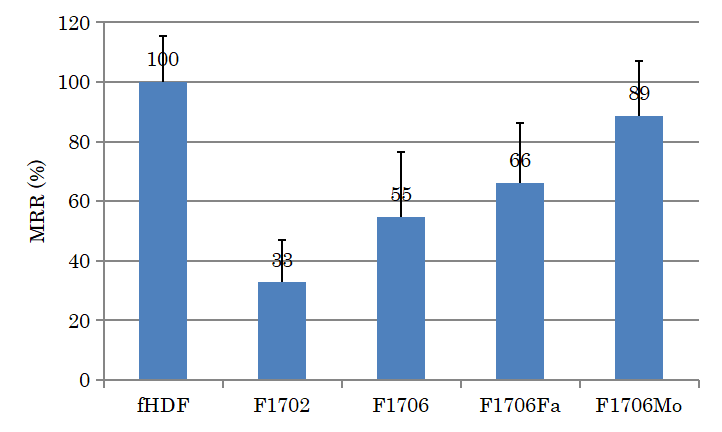

Supplement: Supplementary file 3 [file Data_Sheet_1.doc]
